# Tracking TCRβ Sequence Clonotype Expansions during Antiviral Therapy Using High-Throughput Sequencing of the Hypervariable Region

**DOI:** 10.3389/fimmu.2016.00131

**Published:** 2016-04-05

**Authors:** Mark W. Robinson, Joseph Hughes, Gavin S. Wilkie, Rachael Swann, Stephen T. Barclay, Peter R. Mills, Arvind H. Patel, Emma C. Thomson, John McLauchlan

**Affiliations:** ^1^MRC – University of Glasgow Centre for Virus Research, Institute of Infection, Immunity and Inflammation, University of Glasgow, Glasgow, UK; ^2^School of Biochemistry and Immunology, Trinity Biomedical Sciences Institute, Trinity College Dublin, Dublin, Ireland; ^3^Gartnavel General Hospital, NHS Greater Glasgow and Clyde, Glasgow, UK; ^4^Glasgow Royal Infirmary, NHS Greater Glasgow and Clyde, Glasgow, UK

**Keywords:** T cell receptor, high-throughput sequencing, complementarity determining region 3, hepatitis C virus, *TRBV*, *TBRJ*, clonotype expansion

## Abstract

To maintain a persistent infection viruses such as hepatitis C virus (HCV) employ a range of mechanisms that subvert protective T cell responses. The suppression of antigen-specific T cell responses by HCV hinders efforts to profile T cell responses during chronic infection and antiviral therapy. Conventional methods of detecting antigen-specific T cells utilize either antigen stimulation (e.g., ELISpot, proliferation assays, cytokine production) or antigen-loaded tetramer staining. This limits the ability to profile T cell responses during chronic infection due to suppressed effector function and the requirement for prior knowledge of antigenic viral peptide sequences. Recently, high-throughput sequencing (HTS) technologies have been developed for the analysis of T cell repertoires. In the present study, we have assessed the feasibility of HTS of the TCRβ complementarity determining region (CDR)3 to track T cell expansions in an antigen-independent manner. Using sequential blood samples from HCV-infected individuals undergoing antiviral therapy, we were able to measure the population frequencies of >35,000 TCRβ sequence clonotypes in each individual over the course of 12 weeks. *TRBV*/*TRBJ* gene segment usage varied markedly between individuals but remained relatively constant within individuals across the course of therapy. Despite this stable *TRBV*/*TRBJ* gene segment usage, a number of TCRβ sequence clonotypes showed dramatic changes in read frequency. These changes could not be linked to therapy outcomes in the present study; however, the TCRβ CDR3 sequences with the largest fold changes did include sequences with identical *TRBV*/*TRBJ* gene segment usage and high junction region homology to previously published CDR3 sequences from HCV-specific T cells targeting the HLA-B*0801-restricted ^1395^HSKKKCDEL^1403^ and HLA-A*0101-restricted ^1435^ATDALMTGY^1443^ epitopes. The pipeline developed in this proof of concept study provides a platform for the design of future experiments to accurately address the question of whether T cell responses contribute to SVR upon antiviral therapy. This pipeline represents a novel technique to analyze T cell dynamics in situations where conventional antigen-dependent methods are limited due to suppression of T cell functions and highly diverse antigenic sequences.

## Introduction

The importance of T cell populations as mediators of protective immunity is well documented for a range of viral infections (1–3). To maintain a persistent infection viruses such as hepatitis C virus (HCV) employ a range of mechanisms that subvert these protective T cell responses. These include escape from immune pressure, exhaustion of immune cells, and suppression of immune pathways. Host genetic studies have identified strong associations between specific class I and II human leukocyte antigens (HLA), which present viral peptides to T cells, and spontaneous viral clearance (4, 5). However, the role of T cell responses in individuals with chronic infection is less clear. The reversion of viral escape variants to consensus sequences upon immune suppression highlights the fact that HCV-specific T cells actively exert immune pressure during persistent infection (6). Furthermore, studies have indicated that HCV-specific T cell proliferation and/or interferon (IFN)γ production can predict sustained virological response (SVR) upon treatment, although the evidence for this is conflicting (7–12).

Defining the role of HCV-specific T cells during both chronic infection and antiviral therapy is challenging due to limitations inherent in the experimental assays used to identify HCV-specific T cells. Conventional methods of detecting antigen-specific T cells utilize two basic techniques: (1) antigen stimulation (e.g., ELISpot, proliferation assays, and flow cytometry) and (2) antigen-loaded tetramer staining. Antigen stimulation assays rely on a functional readout of T cell activity, however, chronic HCV infection and exogenous IFNα used during antiviral therapy are both associated with suppressed T cell function. This causes difficulty when assessing T cell responses in these groups of patients. Both antigen stimulation and tetramer staining also rely on prior knowledge of the specific antigenic peptides against which immune responses are directed. For a hugely diverse pathogen such as HCV, this means that even within a single-infected individual, it is unfeasible to profile immune responses targeting all antigenic sequence variants.

**Figure 1:**
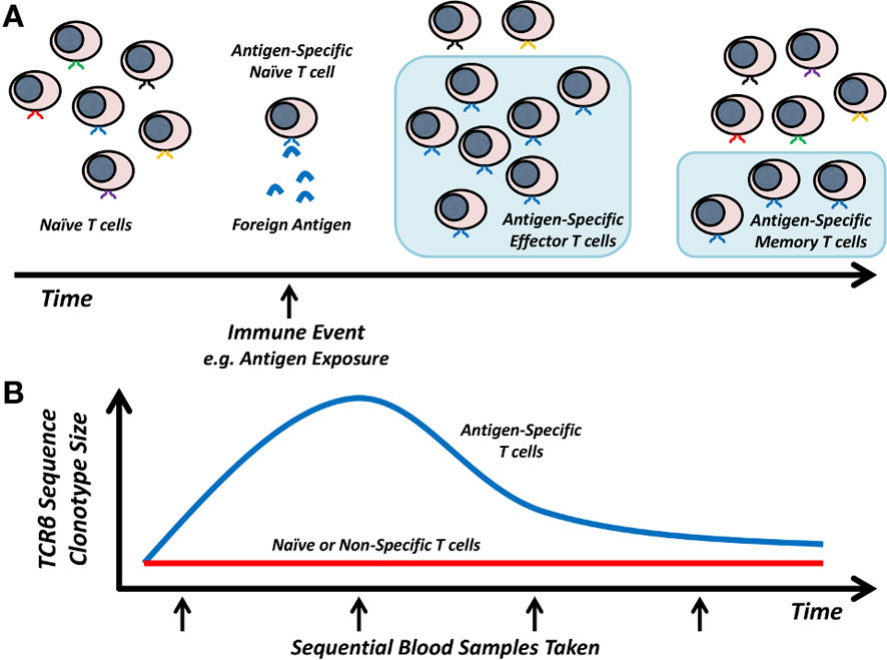
**Model of T cell responses and TCRβ dynamics**. **(A)** Antigen-specific T cell clones expand in response to foreign antigen and then contract to a stable memory T cell population. **(B)** Hypothesized TCRβ sequence clonotype changes in naive/non-specific versus antigen-specific T cells.

During viral infection, antigens from viral proteins, presented by host HLA molecules, are recognized by naive T cells expressing antigen-specific T cell receptors (TCR). This recognition results in T cell activation, which together with co-stimulation signals, leads to the clonal expansion of viral-specific T cells that express cytokines and cytotoxic mediators to facilitate pathogen clearance. Following clearance of the invading pathogen, this clonal population of effector T cells (T_eff_) contracts and a proportion of these cells develop into memory T cell populations, which can persist for a number of years and can rapidly respond upon subsequent re-challenge with the same antigen (13). Throughout this process the antigen-specificity of any particular T cell clone is defined by the unique TCR expressed by the parent naive T cell (as illustrated in Figure 1A). While this is a simplified model of T cell responses, it forms the basis of our theoretical knowledge of how T cells respond to a specific antigen and suggests that it should be possible to utilize sequential TCRβ sequence clonotype size as a proxy for clonal expansion and contraction of antigen-specific T cells during infection in an antigen-independent manner (Figure 1B).

The TCR expressed by conventional T cells is composed of a heterodimer of a single alpha and beta chain (14). This allows recognition of cognate antigen presented by HLA molecules, activating the T cell and inducing the development of an adaptive immune response. During the development and maturation of naive T cells, the TCR gene locus undergoes RAG-mediated recombination of the germline V and J gene segments (in the case of alpha and gamma receptor chains), or V, D, and J gene segments (in the case of beta and delta receptor chains) to produce a functional rearranged TCR genome locus (14, 15). This rearrangement process results in a novel V–J or V–D–J junction region, and gives rise to an estimated theoretical TCRαβ repertoire diversity of 10^18^ in humans, which then undergo thymic selection (16). In the case of the TCRβ chain, the rearrangement involves an estimated 47 possible V regions, two possible D regions, and 13 possible J regions (15). This hypervariable junction region, formed via gene segment rearrangement, is known as the complementarity determining region (CDR)3 and is the most diverse region defining the peptide-binding properties of the TCR.

Recently, high-throughput sequencing (HTS) technologies have been developed for the analysis of CDR3 recombination events, enabling analysis of T cell populations with unprecedented detail. In the present study, we have assessed the feasibility of HTS of the TCRβ CDR3 region to track temporal changes in T cell populations in HCV-infected individuals undergoing antiviral therapy, a setting where conventional T cell assays are limited due to antigenic variation and T cell immune suppression. We describe a pipeline to identify expanding and contracting T cell clones from sequential blood samples and highlight the potential and pitfalls of HTS-based techniques to study T cell responses during infection.

## Materials and Methods

### Clinical Data

Clinical samples were used with informed consent, conforming to the ethical guidelines of the 1975 Declaration of Helsinki and study protocols were approved by the West of Scotland Research Ethics Committee. Blood samples were obtained from chronically HCV-infected patients who were beginning antiviral therapy as part of a larger clinical cohort (17). Blood samples were taken pre-treatment, and at weeks 1, 2, 4, and 12 following the initiation of PEGylated IFNα and ribavirin therapy. Six patients were included in the study: three patients infected with HCV genotype 1 and three patients infected with HCV genotype 3 (as detailed in Table 1). All patients were initially treated with PEGylated IFNα and ribavirin, with the HCV genotype 1-infected individuals also receiving a protease inhibitor from week 4 of therapy. Four patients showed a rapid virologic response (RVR) with viral loads ≤50 IU/mL within 4 weeks, of whom two achieved this reduction within 2 weeks. Two patients (both infected with HCV genotype 1) showed little decline in viral load in response to PEGylated IFNα and ribavirin but cleared the virus by week 12 following the initiation of a protease inhibitor after week 4. All patients successfully cleared the virus following the completion of their treatment and all patients were classified as SVR at 6 months following the end of treatment.

**Table 1:** **Clinical and treatment data**.

	Gender	Age (years)	Source of infection	Gt	Fibroscan (kPa)	IL28B genotype	Previous treatment agent	Previous treatment response	RVR	6-month SVR	PI
Pt 1	M	31	IVDU	3	6.4	CT	N/A	N/A	Y	Y	N/A
Pt 2	M	51	Blood transfusion	1	5.4	CT	IFNα and Ribavirin	Non-responder	N	Y	Boceprevir
Pt 3	F	28	IVDU	3	5.2	nd	N/A	N/A	Y	Y	N/A
Pt 4	M	65	IVDU	1	21.1	CT	IFNα and Ribavirin	Relapse	N	Y	Telaprevir
Pt 5	M	43	IVDU	3	4.8	nd	N/A	N/A	Y	Y	N/A
Pt 6	M	44	IVDU	1	4.5	CT	PEGylated IFNα and Ribavirin	SVR with reinfection	Y	Y	Telaprevir

*Gt, viral genotype; nd, not done; N/A, not applicable*.

### Amplification of the TCR CDR3 Region from Peripheral Blood Mononuclear Cells

DNA-free total RNA was extracted from peripheral blood mononuclear cells (PBMCs) and reverse transcribed using the SuperScript^®^ VILO™ cDNA Synthesis Kit (Invitrogen, Life Technologies, Paisley, UK). RNA quality was assessed on an Agilent 2200 TapeStation (Agilent Technologies, Santa Clara, CA, USA), prior to reverse transcription. To amplify the TCR CDR3 region, a previously published set of primers was utilized consisting of 45 forward primers, each specific to a functional T cell receptor beta variable (*TRBV*) gene segment, and 13 reverse primers, each specific to a T cell receptor beta joining (*TRBJ*) gene segment (18). Duplicate PCR reactions were set up using the QIAGEN^®^ Multiplex PCR kit (Qiagen, Manchester, UK), including 10% Q-solution, and pooled prior to library preparation.

### Library Generation and Sequencing

PCR amplicons were washed, purified, and size-selected using Agencourt^®^ Ampure^®^ XP beads (Beckman Coulter, High Wycombe, UK) and subsequently quantified on a Qubit^®^ 2.0 Fluorometer using the Qubit^®^ dsDNA High Sensitivity kit (Invitrogen). Library preparation, including end repair, A-tailing, and adaptor ligation, was performed using the KAPA HiFi Real-Time PCR Library Amplification kit for Illumina libraries (KAPA Biosystems, Wilmington, MA, USA). The A-tailed PCR amplicons were ligated to NEBNext^®^ Multiplex Oligos for Illumina^®^ (New England Biolabs, Ipswich, MA, USA) and end repair of the forked adaptors was carried out using either Index Primer Set 1 or 2 (New England Biolabs). Adaptor ligated products were quantified using the KAPA SYBR^®^ FAST ABI Prism qPCR library quantification kit (KAPA Biosystems). Sequencing was performed using an Illumina MiSeq™ System Desktop Sequencer (Illumina, San Diego, CA, USA), with a 300 cycle MiSeq version 2 reagent kit (Illumina). To avoid errors due to low sequence diversity within the PCR amplicons the libraries were sequenced with a 25% PhiX spike-in, at a low cluster density (~800 K/mm^2^), and run metrics were checked to ensure that 85–90% of clusters passed quality filtering.

### TCR Sequence Analysis

**Figure 2:**
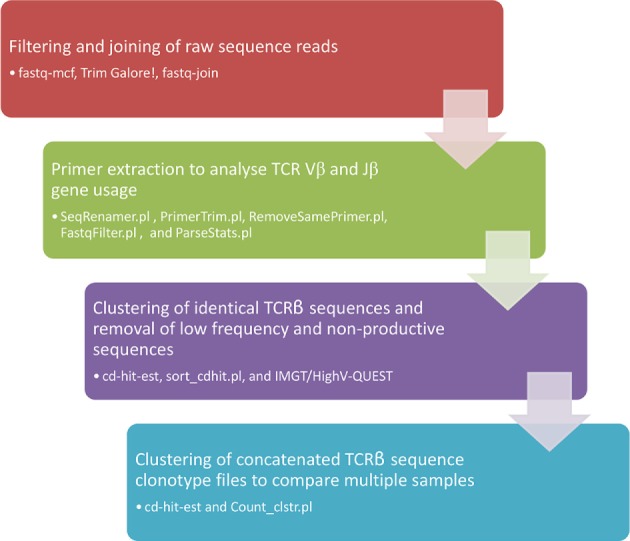
**Flow chart of the sequence analysis pipe-line**.

Raw sequencing reads were trimmed, to remove poor quality sequence and adaptor sequences, using fastq-mcf^1^ and Trim Galore^2^. The paired-end reads were then joined using fastq-join^3^. The remaining filtered and joined sequence reads were analyzed using the pipeline summarized in Figure 2. Briefly, reads were assigned a unique identifier and the *TRBV* and *TRBJ* primer sequences were analyzed using custom perl scripts (SeqRenamer.pl and PrimerTrim.pl). The PrimerTrim.pl script trims the primer sequences from the reads, identifies reads lacking primer sequences, and outputs TCR Vβ and Jβ usage. Sequences lacking a *TRBV* or *TRBJ* primer pair or containing two *TRBV* or *TRBJ* primer sequences were removed using custom perl scripts (RemoveSamePrimer.pl and FastqFilter.pl), and a gene usage text file was generated from the PrimerTrim.pl output (using ParseStats.pl). The .fastq sequencing files were converted to .fasta files and identical reads were grouped into TCRβ sequence clonotypes using cd-hit-est (19). For the cd-hit-est step the global sequence identity threshold was set to 100%, the word size was set at 10, and sequences were aligned in both directions. The output files were parsed using a perl script (sort_cdhit.pl) to extract the number of reads for each TCRβ sequence clonotype, filter on the basis of read frequency and output a representative sequence for each TCRβ sequence clonotype. To explore TCRβ sequence clonotypes between different samples, the output files from the sort_cdhit.pl script from two or more samples were concatenated and analyzed using cd-hit-est a second time. The output files were parsed using a perl script (Count_clstr.pl) to generate a comparison table detailing the TCRβ sequence clonotype number and size for each sample. Representative sequences from non-redundant TCRβ sequence clonotypes, which were represented by more than 0.01% of the total reads (equivalent to a frequency of 0.0001), were analyzed using IMGT/HighV-QUEST (20) to identify sequences with productive CDR3 regions. This cut-off was selected in order to reduce the detection of TCRβ sequence clonotypes that arose due to sequencing error, the frequency of which was estimated from the known primer sequences to range between 0.001 and 0.003 per nucleotide across the sequencing datasets (data not shown). This also had the effect of biasing the TCRβ sequence clonotypes toward expanded T_eff_ and memory T cell populations as opposed to low frequency naive T cell populations. Output files detailing productive sequence frequency for each sample were generated using a perl script (ParseIMGT.pl) and final data normalization and visualization was performed in R. All perl scripts used are publically available at https://github.com/josephhughes/TCRclust.

## Results

### The TCR Repertoire and TRBV/TRBJ Gene Usage Are Relatively Stable within Individuals over the First 12 Weeks of Antiviral Therapy

The results of the TCR sequencing and data processing are summarized in Table 2. Over 400,000 raw paired-end reads were obtained for each sample, which resulted in greater than 300,000 TCR sequences per sample (range 346,336–677,297) after filtering. Following the removal of singleton sequences, which likely represent sequencing/PCR errors, between 35,000 and 115,000 TCRβ sequence clonotypes were detected (Table 2). The number of TCRβ sequence clonotypes varied markedly between patients but was relatively consistent between sampling time points, with patients 2 and 4 both having a low total number of TCRβ sequence clonotypes across all time points sampled (Table 2). These two patients were the oldest in the study (at 51 and 65 years of age), which potentially contributed to the reduced TCRβ repertoire diversity observed. Both individuals also failed to achieve RVR, although both subsequently achieved SVR (Table 1).

**Table 2:** **Sequencing filtering and clustering statistics**.

	Total reads	Joined reads passing filtering	% Reads passing filtering	Singletons	% Singletons	TCRβ sequence clonotypes	Clonotypes >0.0001 read frequency
Pt 1 day 0	712,114	578,962	81.30	138,579	23.94	96,342	351
Pt 1 day 14	634,288	509,817	80.38	123,369	24.20	86,115	309
Pt 1 day 28	678,888	541,744	79.80	131,904	24.35	91,304	273
Pt 1 day 84	729,463	587,775	80.58	129,391	22.01	94,690	351
Pt 2 day 0	757,735	605,308	79.88	59,620	9.85	53,696	649
Pt 2 day 14	780,672	634,618	81.29	53,667	8.46	45,556	735
Pt 2 day 28	719,236	582,503	80.99	48,491	8.32	37,518	889
Pt 2 day 84	794,255	619,977	78.06	48,074	7.75	35,749	1,056
Pt 3 day 0	730,970	568,087	77.72	142,900	25.15	102,441	202
Pt 3 day 14	742,921	603,994	81.30	62,924	10.42	57,993	380
Pt 3 day 28	715,794	591,947	82.70	161,392	27.26	109,160	168
Pt 3 day 84	679,172	535,881	78.90	162,876	30.39	98,310	168
Pt 4 day 0	805,599	636,395	79.00	112,244	17.64	71,884	729
Pt 4 day 14	705,899	568,716	80.57	50,552	8.89	41,283	820
Pt 4 day 28	434,032	346,336	79.80	79,450	22.94	47,085	590
Pt 4 day 84	677,674	528,767	78.03	81,657	15.44	67,809	661
Pt 5 day 0	706,399	572,666	81.07	174,381	30.45	94,164	335
Pt 5 day 14	751,609	606,642	80.71	119,104	19.63	94,296	315
Pt 5 day 28	806,787	641,859	79.56	111,678	17.40	97,188	288
Pt 5 day 84	692,772	545,476	78.74	129,638	23.77	91,356	274
Pt 6 day 0	853,716	660,961	77.42	201,954	30.55	111,499	269
Pt 6 day 14	838,230	666,127	79.47	146,402	21.98	111,654	246
Pt 6 day 28	764,235	630,828	82.54	148,399	23.52	113,641	210
Pt 6 day 84	840,148	677,297	80.62	103,453	15.27	100,277	228

**Figure 3:**
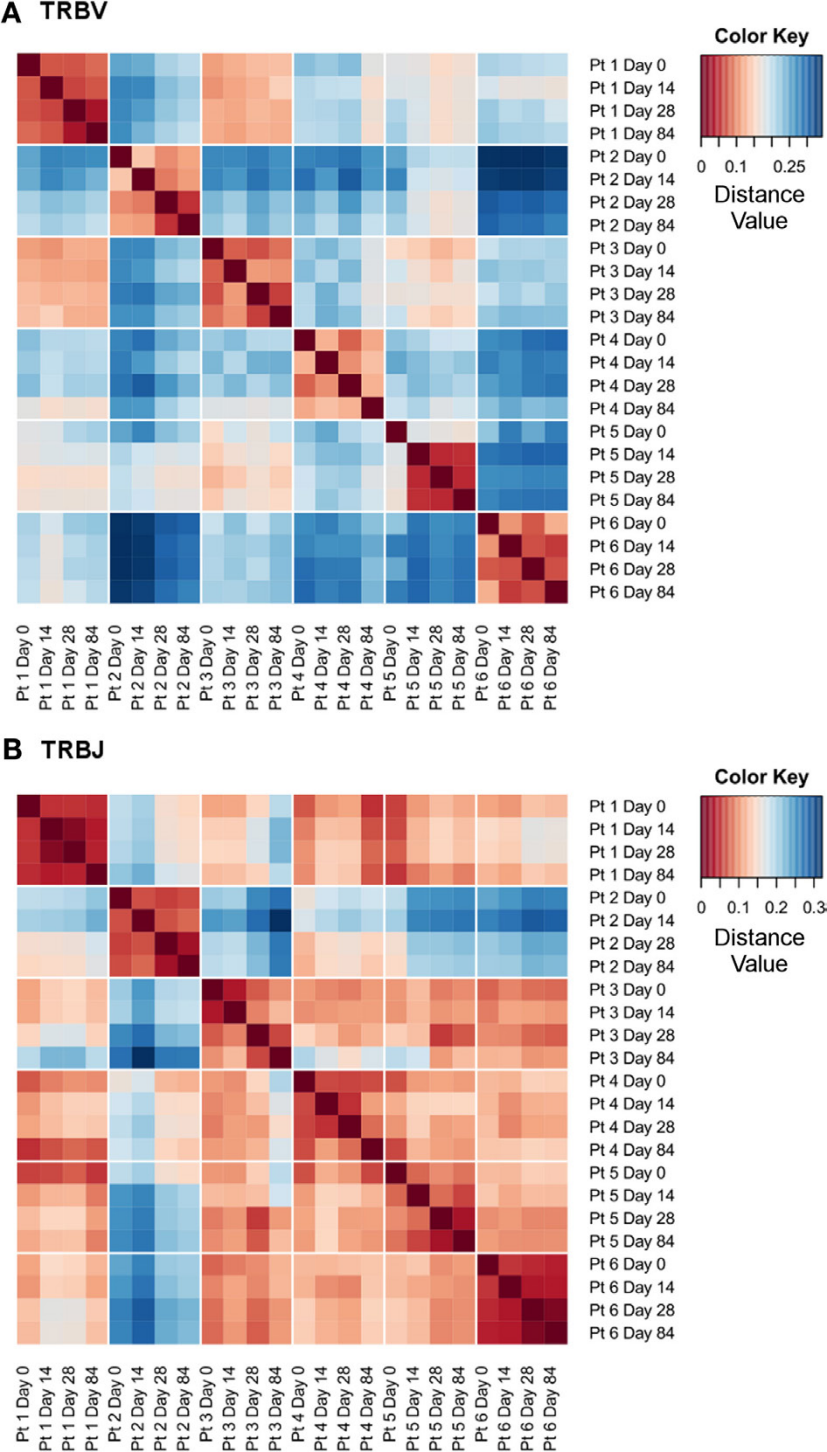
**High intra-individual similarity in *TRBV* and *TRBJ* gene segment usage**. Distance heatmaps using Manhattan distance measures for *TRBV* **(A)** and *TRBJ* **(B)** gene segment usage calculated from the gene segment frequency following the primer trimming step.

In addition to the intra-individual consistency in the total number of TCRβ sequence clonotypes, there was a high degree of intra-individual similarity in terms of gene segment usage. The frequency of *TRBV* and *TRBJ* gene segment usage within sequence datasets, from each individual patient and time point, was used to calculate a distance matrix, using Manhattan distance measures, to provide a metric for the similarity in gene segment usage between datasets, as visualized for *TRBV* in Figure 3A and for *TRBJ* in Figure 3B. The *TRBV* and *TRBJ* gene segment usage was most similar (as indicated by small distance values, visualized as dark red colors) between samples collected at different time points from the same individual. In contrast, the *TRBV* and *TRBJ* gene segment usage was less similar (visualized as a shift toward dark blue colors) between samples from different individuals. The average distance value between intra-individual datasets from different time points for *TRBV* gene segment usage was 0.093 versus 0.221 for the inter-individual datasets (*p* < 0.0001; Mann–Whitney test). The average distance value between intra-individual datasets from different time points for *TRBJ* gene segment usage was 0.059 versus 0.146 for the inter-individual datasets (*p* < 0.0001; Mann–Whitney test). In all six patients the stability of the TCR repertoire, both in terms of numbers of TCRβ sequence clonotypes and of *TRBV*/*TRBJ* gene segment usage, is maintained despite a significant decrease in circulating lymphocytes across the course of therapy (data not shown), as previously identified during IFNα-based treatments (21).

### Mapping Sequential TCRβ Sequence Frequencies Identifies Changing TCRβ Sequence Clonotypes

**Figure 4:**
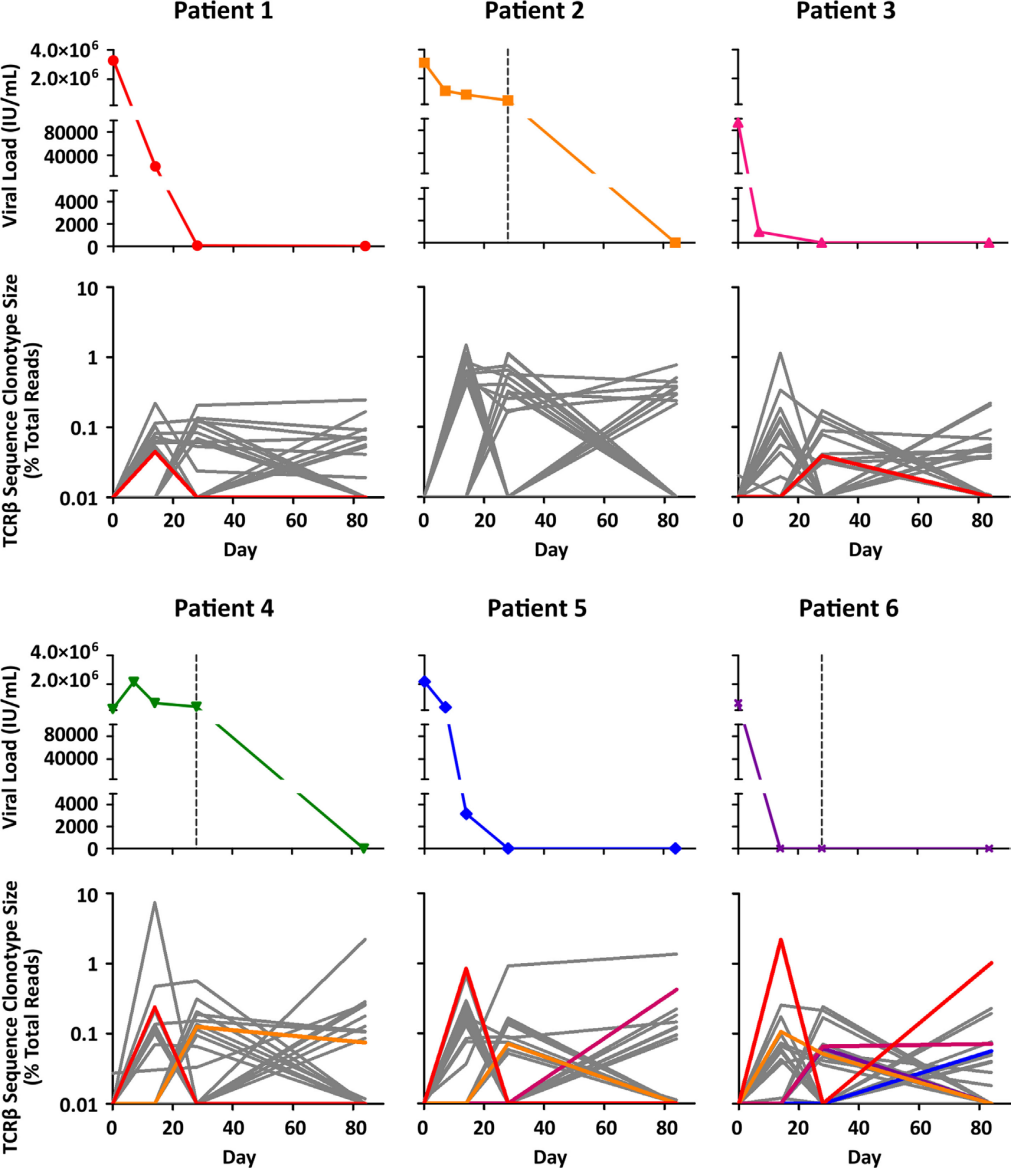
**Viral load kinetics and TCRβ sequence clonotype size during therapy**. For each patient, the upper panel details circulating HCV viral load (IU/mL) across the first 12 weeks of antiviral therapy, while the lower panel shows matched sequential TCRβ sequence clonotype size (expressed as a % of total reads) for the 10 clonotypes showing the greatest expansion at each time-point compared to day 0, from a particular individual. The TCRβ sequence clonotypes highlighted with color have ≥80% identity across the junction region with published HCV-specific TCRβ sequences.

To explore changing T cell populations at a level of detail beyond *TRBV* and *TRBJ* gene segment usage, a pipeline was established to compare productive TCRβ sequence clonotypes within individuals across different time points (Figure 2). In order to avoid detection of TCRβ sequence clonotypes arising due to sequencing error, and to bias our dataset toward expanded T_eff_ and memory T cell populations, we focused on clonotypes with a frequency of >0.0001. While the majority of TCRβ sequence clonotypes had stable read frequencies across the 12 weeks of antiviral therapy, a minority of TCRβ sequence clonotypes showed dramatic changes in read frequency (Figure 4). In certain cases, this represented a fold change in read frequency of hundreds for individual TCRβ sequence clonotypes which either expanded or contracted between sequential time points. These dramatic changes within specific TCRβ sequence clonotypes are not apparent at the level of TCRβ gene segment usage (Figure 3) or the total number of TCRβ sequence clonotypes detected (Table 2).

Comparing the four patients with RVR upon PEGylated IFNα and ribavirin therapy versus the two patients (patients 2 and 4) who only showed a reduction in viral load upon initiation of protease inhibitor treatment at week 4, a trend toward increased TCRβ sequence clonotype size was noted (Figure 4). The significance of this observation in regard to RVR is unclear. Patients 2 and 4 were the oldest and had lower total numbers of TCRβ sequence clonotypes across all time points, which contributed to an increase in the size of individual clonotypes when expressed as a % of total reads. This limitation in the data highlights one of the difficulties in directly comparing TCRβ repertoires between patients and necessitates larger, age-matched patient groups in future studies.

The temporal nature of the TCRβ sequence data allows it to be compared to clinical changes over the course of antiviral therapy, e.g., viral load, as shown in Figure 4. This facilitates the identification of TCRβ sequence clonotypes that show frequency changes concurrent with a decline in HCV viral load. For patients 3, 5, and 6, dramatic declines in HCV viral load are associated with the large expansions of particular TCRβ sequence clonotypes (Figure 4). In these three patients, the maximum TCRβ sequence clonotype fold increase between day 0 and day 14 was 114×, 83×, and 220×, respectively. The association with declines in viral load suggests that these TCRβ sequence clonotypes may be relevant for viral control in these patients and distinguishes these clonotypes from others, which show even larger expansions, e.g., the TCRβ sequence clonotype in patient 4 that expands 742× between day 0 and day 14 (Figure 4).

To explore the possible antigen-specificity of expanding TCRβ sequence clonotypes identified in Figure 4, we interrogated sequence databases containing information on experimentally validated antigen-specific TCRβ sequences. Using BLAST searches (blastp) of non-redundant human protein sequences, we identified 12 TCRβ sequence clonotypes with ≥80% identity to published HCV-specific TCRβ CDR3 sequences (Table 3). These HCV-specific TCRβ CDR3 sequences are specific for either the HLA-B*0801-restricted ^1395^HSKKKCDEL^1403^ epitope or the HLA-A*0101-restricted, ^1435^ATDALMTGY^1443^ epitope (22). Three of the 12 TCRβ sequence clonotypes with ≥80% identity to HCV-specific TCRβ CDR3 sequences had matching *TRBV* and *TRBJ* gene segment usage (Table 3) and highly similar junction regions, differing by two (clonotype 3765) or three amino acids (clonotypes 10041, and 11938). This provides additional evidence that the expanding TCRβ sequence clonotypes detected in the present study are HCV-specific although in the absence of functional validation this is impossible to definitively prove.

**Table 3:** **Expanding TCRβ sequence clonotypes with ≥80% identity to published HCV-specific TCRβ sequences**.

Clonotype (patient)	Day detected	Fold increase	*TRBV*	*TRBJ*	Junction sequence	Matched accession	% Identity	Matched *TRBV*/*TRBV*
670 (pt 1)	14	4.5	7–6, 7–7, or 7–9	2–2	CASSQSQTGNTGELFF	ADQ19362.1	81	No
3765 (pt 3)	28	3.9	10–3	2–3	CAISTSGRGTDTQYF	ADQ19287.1	87	Yes
9961 (pt 4)	14	23.9	19	2–6	CASSISDSGANVLTF	ADQ19329.1	80	No
9052 (pt 4)	28	12.5	28	2–7	CASSLGSGTTYEQYF	ADQ19416.1	80	No
2026 (pt 5)	14	83.8	6–1, 6–5, or 6–8	2–2	CASRTGSSDTGELFF	ADQ19381.1	87	No
6896 (pt 5)	28	7.1	7–6	2–2	CASSSRTGVNTGELFF	ADQ19362.1	88	No
10020 (pt 5)	84	42.0	6–1	2–2	CASRTGSSDTGELFF	ADQ19381.1	87	No
372 (pt 6)	14, 84	220.5, 102.5	7–2	1–1	CASSDRDGGWTEAFF	ADQ19336.1	80	No
8261 (pt 6)	14, 28	10.7, 5.2	19	1–1 or 1–5	CASSIDQGGNQPQHF	ADQ19349.1	80	No
12872 (pt 6)	28, 84	6.6, 7.1	5–6	1–3	CASSLSAGDQPQHF	ADQ19293.1	80	No
10041 (pt 6)	28	6.2	19	1–5	CASSIRQARTQPQHF	ADQ19349.1	80	Yes
11938 (pt 6)	84	5.7	19	1–5	CASSIDQGGNQPQHF	ADQ19349.1	80	Yes

## Discussion

High-throughput sequencing technologies are revolutionizing our understanding of T cell biology. The ability to detect and sequence thousands of TCR sequences simultaneously has provided detailed understanding of the clonal T cell population structure in both health and disease. In this context, techniques allowing the integration of TCR sequence data from sequential time points provide the possibility to track clonal T cell populations over time and identify responding T cell populations in the absence of prior knowledge of antigen-specificity. This is particularly relevant in human diseases where the specific antigen driving T cell activation is unknown (e.g., autoimmunity) or the nature of the infectious pathogen results in high inter- and intrahost antigenic variation (e.g., hepatitis C virus infection). It is also valuable in settings (such as HCV treatment) where T cell numbers and function are suppressed. In the present study, we have developed a pipeline for the integration of sequential TCR HTS data, allowing analysis of multiple sequential datasets each containing over half a million sequence reads.

Numerous TCRβ sequence clonotypes expanded and contracted over the first 12 weeks of therapy, despite a lack of gross changes in *TRBV* and *TRBJ* gene usage over the course of HCV antiviral therapy, and the overall stability of the TCRβ repertoire within individuals. The clonotype-specific nature of this T cell response may contribute to the conflicting data regarding the importance of HCV-specific T cell responses during HCV therapy (7–12). Diverse T cell repertoires can predict good disease outcomes in mice infected with herpes simplex virus-1 (23); however, it is unclear if this also applies in human disease. A more diverse T cell response potentially allows for the selection of high avidity T cell clones and limits pathogen immune escape (24). In HCV infection, T cell responses are critical in controlling infection, as evidenced by class I and II HLA associations (25–28), CD4^+^ and CD8^+^ depletion experiments in the chimpanzee model (29–31) and functional assays during early HCV infection (32). Studies during acute HCV infection have shown by ELISpot and flow cytometry that the T cell response during spontaneous clearance is broad, targets multiple antigens and must be sustained for HCV to be eliminated during early infection. However, only a single study has sequenced TCR repertoires from functionally validated HCV-specific T cells, and this only investigated two specific antigenic peptides (22). This small number of validated HCV-specific TCRβ CDR3 sequences limits the inferences that can be made using a sequence similarity approach as employed in the present study. In addition, this similarity approach does not prove that the identified TCRβ sequence clonotypes are HCV-specific, due to the influence that TCRα pairing and HLA-restriction have on antigen recognition. This limitation in the present study highlights the need to integrate HTS techniques with the isolation of antigen-specific T cell populations to enable validation of antigen-specific TCR sequences.

Conventional studies of T cell responses during treatment are hampered by low numbers of T cells and reduced cytokine production, issues avoided when using an HTS approach. Evidence of a restoration of HCV-specific T cell populations over the course of HCV therapy has recently been obtained in patients treated with IFN-free regimes, where individuals who achieved SVR had an increased proliferation of HCV-specific CD8^+^ T cells within 4 weeks of starting therapy (33). Due to a reliance on HCV-specific tetramers, this study was limited to the analysis of T cells specific for one of four HCV peptides, highlighting the limitations imposed by current methodologies. Utilizing HTS of TCR sequences in a larger number of patients could help to answer fundamental questions on the role of the immune response in viral control during and immediately after treatment. It may also be of use in acute infection, where many current studies require live cells to be available from early in infection – a stage which is usually asymptomatic. HTS of TCR sequences has the potential to be carried out on stored blood samples without the need for PBMC separation. In conjunction with functional validation experiments, this would allow for a comprehensive analysis of T cell responses in a larger number of infected individuals.

The present proof of concept study highlights the large inter-individual variation detected in TCRβ sequence clonotype structures, which hampers comparisons between individuals in this sort of observational study design. In particular, patients 2 and 4, the two oldest patients in the present study, showed a lower total number of TCRβ sequence clonotypes, and a greater proportion of TCRβ sequence clonotypes with read frequencies >0.0001. This altered repertoire, with evidence of TCRβ sequence clonotype expansion, is similar to the T cell repertoire changes observed in aging individuals with human cytomegalovirus (HCMV) infection (34, 35). Future studies should utilize large cohorts of age- and HLA-matched individuals with defined treatment outcomes to account for inter-individual variation and accurately address the question of whether T cell responses contribute to SVR upon antiviral therapy. The pipeline developed in the analysis presented here provides a platform for the design of these experiments.

To avoid sampling or sequencing biases that may have hindered the identification of T cell expansion or contraction, we included the use of pooled PCR reactions and a high read frequency cut-off for the analysis of TCRβ sequence clonotypes. The use of unique molecular identifiers has been recently introduced to limit the effects of PCR bias in HTS experiments and this technique offers significant advantages to the analysis of future HTS studies of the TCR locus (36, 37). Sampling bias – arising from the fact that we can only sample a fraction of an individual’s total T cell pool – is more difficult to address. This issue is of particular relevance for low frequency T cell clones and maintaining a high read frequency cut-off can help to avoid sampling bias. Sampling bias is well recognized in the field of population ecology where a number of techniques have been developed to control for these biases, and the application of these methods to HTS should be expanded in the future (37, 38).

In summary, the analysis of T cell responses through HTS techniques has the potential to greatly expand our understanding of the role of T cells in health and disease. The development of sequential analysis of TCRβ sequence clonotypes can provide an antigen-independent view of T cell dynamics in complex tissue environments, complementing existing T cell phenotyping assays, and providing insights into the role of immunological diversity in protection from infectious disease.

## Author Contributions

MR contributed to study concept and design, acquisition and analysis of data, and wrote the manuscript. JH developed the bioinformatics pipeline and contributed to data analysis and manuscript writing. GW contributed technical and experimental support. RS, SB, and PM contributed to patient recruitment and administrative support. AP contributed to study supervision and funding. ET and JM contributed to study design, supervision, funding, and manuscript review.

## Conflict of Interest Statement

The authors declare that the research was conducted in the absence of any commercial or financial relationships that could be construed as a potential conflict of interest.

## Footnotes

^1^https://code.google.com/p/ea-utils/wiki/FastqMcf

^2^http://www.bioinformatics.babraham.ac.uk/projects/trim_galore/

^3^https://code.google.com/p/ea-utils/wiki/FastqJoin

## Abbreviations

CDR, complementarity determining region; HLA, human leukocyte antigen; HTS, high-throughput sequencing; IFN, interferon; PBMC, peripheral blood mononuclear cells; TCR, T cell receptor; Teff, effector T cells; SVR, sustained virological response.

## References

[1] Park S-H Rehermann B. Immune responses to HCV and other hepatitis viruses. Immunity (2014) 40:13–24. 10.1016/j.immuni.2013.12.010 24439265 PMC4480226

[2] Walker BD Yu XG. Unravelling the mechanisms of durable control of HIV-1. Nat Rev Immunol (2013) 13:487–98. 10.1038/nri3478 23797064

[3] La Gruta NL Turner SJ. T cell mediated immunity to influenza: mechanisms of viral control. Trends Immunol (2014) 35:396–402. 10.1016/j.it.2014.06.004 25043801

[4] Fitzmaurice K Hurst J Dring M Rauch A McLaren PJ Günthard HF Additive effects of HLA alleles and innate immune genes determine viral outcome in HCV infection. Gut (2015) 64:813–9. 10.1136/gutjnl-2013-306287 24996883 PMC4392199

[5] McKiernan SM Hagan R Curry M McDonald GSA Kelly A Nolan N Distinct MHC class I and II alleles are associated with hepatitis C viral clearance, originating from a single source. Hepatology (2004) 40:108–14. 10.1002/hep.20261 15239092

[6] Honegger JR Kim S Price AA Kohout JA McKnight KL Prasad MR Loss of immune escape mutations during persistent HCV infection in pregnancy enhances replication of vertically transmitted viruses. Nat Med (2013) 19:1529–33. 10.1038/nm.3351 24162814 PMC3823809

[7] Larrubia J-R Lokhande M-U Moreno-Cubero E García-Garzón S Miquel J Parra-Cid T HCV-specific CD8+ cell detection at week 12 of chronic hepatitis C treatment with PEG-interferon-α2b/ribavirin correlates with infection resolution. Cell Immunol (2013) 286:31–8. 10.1016/j.cellimm.2013.11.001 24287274

[8] Barnes E Gelderblom HC Humphreys I Semmo N Reesink HW Beld MG Cellular immune responses during high-dose interferon-alpha induction therapy for hepatitis C virus infection. J Infect Dis (2009) 199:819–28. 10.1086/597072 19434929

[9] Caetano J Martinho A Paiva A Pais B Valente C Luxo C. Differences in hepatitis C virus (HCV)-specific CD8 T-cell phenotype during pegylated alpha interferon and ribavirin treatment are related to response to antiviral therapy in patients chronically infected with HCV. J Virol (2008) 82:7567–77. 10.1128/JVI.02175-07 18480446 PMC2493325

[10] Pilli M Zerbini A Penna A Orlandini A Lukasiewicz E Pawlotsky J-M HCV-specific T-cell response in relation to viral kinetics and treatment outcome (DITTO-HCV project). Gastroenterology (2007) 133:1132–43. 10.1053/j.gastro.2007.06.059 17919489

[11] Cramp ME Rossol S Chokshi S Carucci P Williams R Naoumov NV. Hepatitis C virus-specific T-cell reactivity during interferon and ribavirin treatment in chronic hepatitis C. Gastroenterology (2000) 118(2):346–55. 10.1016/S0016-5085(00)70217-4 10648463

[12] Aberle JH Perstinger G Weseslindtner L Sinzinger U Gurguta C Steindl-Munda P CD4+ T cell responses in patients with chronic hepatitis C undergoing peginterferon/ribavirin therapy correlate with faster, but not sustained, viral clearance. J Infect Dis (2007) 195:1315–9. 10.1086/513278 17397001

[13] Farber DL Yudanin NA Restifo NP. Human memory T cells: generation, compartmentalization and homeostasis. Nat Rev Immunol (2014) 14:24–35. 10.1038/nri3567 24336101 PMC4032067

[14] Davis MM Bjorkman PJ. T-cell antigen receptor genes and T-cell recognition. Nature (1988) 334:395–402. 10.1038/334395a0 3043226

[15] van Dongen JJ Langerak AW Brüggemann M Evans PA Hummel M Lavender FL Design and standardization of PCR primers and protocols for detection of clonal immunoglobulin and T-cell receptor gene recombinations in suspect lymphoproliferations: report of the BIOMED-2 concerted action BMH4-CT98-3936. Leukemia (2003) 17:2257–317. 10.1038/sj.leu.2403202 14671650

[16] Attaf M Huseby E Sewell AK. αβ T cell receptors as predictors of health and disease. Cell Mol Immunol (2015) 12:391–9. 10.1038/cmi.2014.134 25619506 PMC4496535

[17] Robinson MW Swann R Sigruener A Barclay ST Mills PR McLauchlan J Elevated interferon-stimulated gene transcription in peripheral blood mononuclear cells occurs in patients infected with genotype 1 but not genotype 3 hepatitis C virus. J Viral Hepat (2014) 22:384–90. 10.1111/jvh.12310 25200131 PMC4409080

[18] Robins HS Campregher PV Srivastava SK Wacher A Turtle CJ Kahsai O Comprehensive assessment of T-cell receptor beta-chain diversity in alphabeta T cells. Blood (2009) 114:4099–107. 10.1182/blood-2009-04-217604 19706884 PMC2774550

[19] Li W Godzik A. Cd-hit: a fast program for clustering and comparing large sets of protein or nucleotide sequences. Bioinformatics (2006) 22:1658–9. 10.1093/bioinformatics/btl158 16731699

[20] Li S Lefranc M-P Miles JJ Alamyar E Giudicelli V Duroux P IMGT/HighV QUEST paradigm for T cell receptor IMGT clonotype diversity and next generation repertoire immunoprofiling. Nat Commun (2013) 4:2333. 10.1038/ncomms3333 23995877 PMC3778833

[21] Dieterich DT Spivak JL. Hematologic disorders associated with hepatitis C virus infection and their management. Clin Infect Dis (2003) 37:533–41. 10.1086/376971 12905138

[22] Miles JJ Thammanichanond D Moneer S Nivarthi UK Kjer-Nielsen L Tracy SL Antigen-driven patterns of TCR bias are shared across diverse outcomes of human hepatitis C virus infection. J Immunol (2011) 186:901–12. 10.4049/jimmunol.1003167 21160049

[23] Messaoudi I Guevara Patiño JA Dyall R LeMaoult J Nikolich-Zugich J. Direct link between mhc polymorphism, T cell avidity, and diversity in immune defense. Science (2002) 298:1797–800. 10.1126/science.1076064 12459592

[24] Turner SJ La Gruta NL Kedzierska K Thomas PG Doherty PC. Functional implications of T cell receptor diversity. Curr Opin Immunol (2009) 21:286–90. 10.1016/j.coi.2009.05.004 19524428 PMC2706259

[25] Neumann-Haefelin C Timm J Schmidt J Kersting N Fitzmaurice K Oniangue-Ndza C Protective effect of human leukocyte antigen B27 in hepatitis C virus infection requires the presence of a genotype-specific immunodominant CD8+ T-cell epitope. Hepatology (2010) 51:54–62. 10.1002/hep.23275 20034048 PMC4396188

[26] Kim AY Kuntzen T Timm J Nolan BE Baca MA Reyor LL Spontaneous control of HCV is associated with expression of HLA-B 57 and preservation of targeted epitopes. Gastroenterology (2011) 140:686–96. 10.1053/j.gastro.2010.09.042 20875418 PMC3021586

[27] Thursz M Yallop R Goldin R Trepo C Thomas HC Influence of MHC class II genotype on outcome of infection with hepatitis C virus. The HENCORE group. Hepatitis C European Network for Cooperative Research. Lancet (2016) 354:2119–24. 10.1016/S0140-6736(99)91443-5 10609818

[28] Barrett S Ryan E Crowe J. Association of the HLA-DRB1*01 allele with spontaneous viral clearance in an Irish cohort infected with hepatitis C virus via contaminated anti-D immunoglobulin. J Hepatol (1999) 30:979–83. 10.1016/S0168-8278(99)80249-9 10406173

[29] Woollard DJ Grakoui A Shoukry NH Murthy KK Campbell KJ Walker CM. Characterization of HCV-specific Patr class II restricted CD4+ T cell responses in an acutely infected chimpanzee. Hepatology (2003) 38:1297–306. 10.1053/jhep.2003.50478 14578870

[30] Cooper S Erickson AL Adams EJ Kansopon J Weiner AJ Chien DY Analysis of a successful immune response against hepatitis C virus. Immunity (1999) 10:439–49. 10.1016/S1074-7613(00)80044-8 10229187

[31] Grakoui A Shoukry NH Woollard DJ Han J-H Hanson HL Ghrayeb J HCV persistence and immune evasion in the absence of memory T cell help. Science (2003) 302:659–62. 10.1126/science.1088774 14576438

[32] Cox AL Mosbruger T Lauer GM Pardoll D Thomas DL Ray SC. Comprehensive analyses of CD8+ T cell responses during longitudinal study of acute human hepatitis C. Hepatology (2005) 42:104–12. 10.1002/hep.20749 15962289 PMC2759395

[33] Martin B Hennecke N Lohmann V Kayser A Neumann-Haefelin C Kukolj G Restoration of HCV-specific CD8+ T cell function by interferon-free therapy. J Hepatol (2014) 61:538–43. 10.1016/j.jhep.2014.05.043 24905492

[34] Sylwester AW Mitchell BL Edgar JB Taormina C Pelte C Ruchti F Broadly targeted human cytomegalovirus-specific CD4+ and CD8+ T cells dominate the memory compartments of exposed subjects. J Exp Med (2005) 202:673–85. 10.1084/jem.20050882 16147978 PMC2212883

[35] Weekes MP Wills MR Mynard K Hicks R Sissons JG Carmichael AJ. Large clonal expansions of human virus-specific memory cytotoxic T lymphocytes within the CD57+ CD28- CD8+ T-cell population. Immunology (1999) 98:443–9. 10.1046/j.1365-2567.1999.00901.x 10583606 PMC2326947

[36] Egorov ES Merzlyak EM Shelenkov AA Britanova OV Sharonov GV Staroverov DB Quantitative profiling of immune repertoires for minor lymphocyte counts using unique molecular identifiers. J Immunol (2015) 194:6155–63. 10.4049/jimmunol.1500215 25957172

[37] Greiff V Miho E Menzel U Reddy ST. Bioinformatic and statistical analysis of adaptive immune repertoires. Trends Immunol (2015) 36:738–49. 10.1016/j.it.2015.09.006 26508293

[38] Laydon DJ Bangham CRM Asquith B. Estimating T-cell repertoire diversity: limitations of classical estimators and a new approach. Philos Trans R Soc Lond B Biol Sci (2015) 370:1675. 10.1098/rstb.2014.0291 26150657 PMC4528489

